# “Post-lockdown Depression”: Adaptation Difficulties, Depressive Symptoms, and the Role of Positive Solitude When Returning to Routine After the Lifting of Nation-Wide COVID-19 Social Restrictions

**DOI:** 10.3389/fpsyt.2022.838903

**Published:** 2022-03-11

**Authors:** Shoshi Keisari, Yuval Palgi, Lia Ring, Adi Folkman, Boaz M. Ben-David

**Affiliations:** ^1^School of Creative Arts Therapies, University of Haifa, Haifa, Israel; ^2^The Centre for Research and Study of Aging, University of Haifa, Haifa, Israel; ^3^The Emily Sagol Creative Arts Therapies Research Center, University of Haifa, Haifa, Israel; ^4^Department of Gerontology, University of Haifa, Haifa, Israel; ^5^Department of Counseling and Human Development, University of Haifa, Haifa, Israel; ^6^Baruch Ivcher School of Psychology, Reichman University (IDC), Herzliya, Israel; ^7^Department of Speech-Language Pathology, University of Toronto, Toronto, ON, Canada; ^8^Knowledge, Innovation, Talent, Everywhere (KITE), Toronto Rehabilitation Institute, University Health Networks, Toronto, ON, Canada

**Keywords:** depressive symptoms, adaptation difficulties, positive solitude, mid-life, older adults, lockdown

## Abstract

**Objectives:**

The aim of the current study was to identify difficulties in adapting to normal life once COVID-19 lockdown has been lifted. Israel was used as a case study, as COVID-19 social restrictions, including a nation-wide lockdown, were lifted almost completely by mid-April 2021, following a large-scale vaccination operation.

**Methods:**

A sample of 293 mid-age and older Israeli adults (*M* age = 61.6 ± 12.8, range 40–85 years old) reported on return-to-routine adaptation difficulties (on a novel index), depression, positive solitude, and several demographic factors.

**Results:**

Of the participants, 40.4% met the criteria of (at least) mild depressive symptoms. Higher levels of adaptation difficulties were related to higher ratios of clinical depressive symptoms. This link was moderated by positive solitude. Namely, the association between return-to-routine adaptation difficulties and depression was mainly indicated for individuals with low positive solitude.

**Conclusions:**

The current findings are of special interest to public welfare, as adaptation difficulties were associated with higher chance for clinical depressive symptoms, while positive solitude was found to be as an efficient moderator during this period. The large proportion of depressive symptoms that persist despite lifting of social restrictions should be taken into consideration by policy makers when designing return-to-routine plans.

## Introduction

The response to the COVID-19 pandemic has led to unprecedented social distancing measures worldwide. These included lockdowns, where individuals were ordered to stay at home for weeks (1). These restrictions were found to yield psychological distress for many, with various aspects of mental health disturbance, including inflated rates of depressive symptoms, occurring across populations (2, 3). In response to the pandemic, global efforts have been made to vaccinate entire populations to lessen mortality and lift social restrictions (4, 5). Paradoxically, however, achieving the return to normal life may itself have a cost. Although many people waited for the restrictions to be lifted, these same restrictions provided a respite and changed the life course of many individuals who may experience adaptation difficulties and depressive symptoms during the transition period to their former routine. We hypothesize that the long-term effects of social restrictions may be also manifested when people are faced with a return to daily routines and obligations.

Although social restrictions helped mitigate the spread of the virus, they had severe psychological consequences. Social distancing incurred significant life changes that could be experienced as negative or positive, such as losing or changing jobs and un/healthy lifestyle changes (6). Restrictions also severely disrupted social interactions, social presence, communication and daily routines, all important to maintain cognitive performance and wellbeing [see (7, 8)]. Taken together, social restrictions have been found to impair mental health, including an increase in anxiety, depressive symptoms, loneliness and social isolation (9–11). The current study focuses on depressive symptoms when returning to routine following the release from COVID-19 lockdown.

Depressive disorders are usually conceptualized along a continuum, progressing from mild to moderate to severe, characterized by the duration and severity of the symptoms (12). Moderate and severe depression are leading causes for disability, with greater stability and a higher risk for suicidality (13). However, mild symptoms are still considered as a serious medical condition that leads to professional and personal disabilities, social problems and reduced quality of life (14, 15). Mild depression often represents a maladaptive response of the individual to environmental stressors and is frequently prodromal to major depression disorders (16). Note, mild depression is different than normal sadness, as based on the number, duration and quality of presented symptoms, and can be diagnosed by linguistic indicators (17). Recent literature shows that since the COVID-19 outbreak, the prevalence of depressive symptoms increased among the adult population worldwide (2). For example, in 2020 ~24.6% of adults in the USA experienced mild depressive symptoms vs. 16.2% before the pandemic. A similar trend was also noted for moderate depressive symptoms, with an increase from 5.7% before the pandemic to 14.8% for US adults [(18, 19); for European samples, see (20, 21)].

Demographic characteristics have been found to have a large impact on the extent of the effects of COVID-19 social restriction (11, 22). For example, a higher prevalence of depression and anxiety symptoms were indicated for women, and for people who are not partnered. In addition, caregivers who must adapt their work routines to care for others at home were at a higher risk of psychological burden (23). Middle-aged adults appear to be more susceptible to experiencing symptoms of mental illness during the pandemic, as compared to older adults (11). In the current study, we examine the possible contribution of these demographic characteristics to depressive symptoms, following the termination of a COVID-19 lockdown.

Coping strategies, skills and personality traits were also associated with the impact of the pandemic (24). For example, centrality appraisals and planning, controllability appraisals, as well as coping strategies were related to the differences in subjective wellbeing among adults during the early stage of the pandemic (25). High levels of arts engagement constituted a potent buffer against subsequent COVID-19 anxiety (26, 27). Personality traits of neuroticism and extroversion were also associated with mental health during the COVID-19 pandemic: neuroticism negatively related and extroversion positively related to mental health (28).

In the current study, we focus on positive solitude—the volitional positive experience while being by oneself (29)—as a possible moderator for the negative effects of lockdown. Positive solitude is defined as the choice to dedicate time to a meaningful, enjoyable activity or experience conducted by oneself. This activity/experience might be spiritual, functional, recreational or of any chosen type, and it is independent of any external or physical conditions (30). It is not surprising to find that positive solitude has been identified as a source for resilience during social restrictions (31). In general, it is associated with wellbeing and better emotion regulation and introspection (32, 33). Moreover, a high capacity of solitude was associated with low levels of depression (34) and post-traumatic stress symptoms (35). During COVID-19 social restrictions, it was found that the preference for solitude (in tandem with other personality variables) predicted individuals' improved mental health and creativity. Namely, people who experience themselves as more stable when they are alone expressed a lower level of loneliness and performed better than their peers on a creative insight task (31).

As aforementioned, numerous studies have examined the effects of social restrictions on mental health. However, no study to date has directly explored the negative impacts of returning to normal life after social restrictions have been lifted. Nevertheless, the literature points to the possible negative impacts of returning to routine. For example, during the first COVID-19 wave, Europeans expressed negative expectations regarding the future and return to normal life, fears of an economic depression, and concerns regarding dangers to freedom (36). A study in our lab showed that even after COVID-19 vaccinations, mental health symptoms were not alleviated (5). Indeed, even the release from incarceration, a much stronger form of restriction, incurs post-prison adaptation difficulties and psychological symptoms (37). Paradoxically, as social restrictions can be experienced as a break from work life (6) and even relate to improved wellbeing for some individuals (38, 39), the difficulties involved in return-to-routine when they are lifted can be related to difficulties adapting to daily work when returning from vacation (40).

In the current study, our main aim was to identify difficulties in adapting to normal life once a nation-wide COVID-19 lockdown (imposed on the whole population) was lifted during April 2021 in Israel. Our second aim was to test whether a higher level of return-to-routine adaptation difficulties is associated with higher depressive symptoms. Our third aim was to test whether this link is moderated by positive solitude, after controlling for individual demographic characteristics (gender, age, SES and family status).

Israel constitutes a special case study, as it was one of the first countries to initiate a large-scale vaccination operation (41), with social restrictions lifted almost completely by mid-April 2021. Israel is also unique as every Israeli citizen is entitled to healthcare services under the National Health Insurance Law. This was at the base of the success of the early vaccination campaign that lead to a quick nation-wide (rather than regional) release from lockdown and other social restrictions, and full re-opening of schools for the first time in over a year, at the end of the COVID-19 third wave (41, 42).

## Methods

### Participants and Procedure

Data from 293 Israeli adults (age ≥ 40) were collected *via* social media platforms from April 12 to May 3, 2021 (342 individuals replied and 49 individuals did not complete the survey). By April 12, the first day of distribution of the questionnaire, 57.3% of the population had received the first dose of the vaccination. Only 225 Israelis tested positive for COVID-19 on that day, and the Israeli government announced a return to routine including the re-opening of schools, workplaces and shopping centers (41).

Data were obtained using a convenience sample of 293 Israelis [*M* age = 61.57, *SD* = 12.81, range (40–85) years old]. Most of them were women (*n* = 222, 75.8%), married or cohabitating (*n* = 232, 79.2%). Socio economic status was self-reported as *M* = 3.88, *SD* = 0.88 (on a scale ranged from 1 = “*Not good at all”* to 5 = “*Very good”*). All participants were informed about the subject of the research and electronically provided their informed consent to participate. Ethical approval was received from the Institutional Review Board of Reichman University, Herzliya.

### Measures

Participants completed a demographic questionnaire, including age, gender, marital status as well as economic status.

#### Depression

Depression was assessed using the 9-item Patient Health Questionnaire-9 (PHQ-9). Participants were asked: “Over the last 2 weeks, how often have you been bothered by the following problems?” An example of a problem is: “Little interest or pleasure in doing things.” Items were rated on a scale of 0 (Not at all) to 3 (Nearly every day). In this study, the Cronbach's coefficient was α = 0.837.

#### Positive Solitude

Positive Solitude was assessed by the 9-item Positive Solitude Scale (43). An example of an item is: “When I find time for myself, I succeed better at making future plans.” Items were rated on a scale ranging from 1 (Not at all) to 5 (Most of the time). In this study, the Cronbach's coefficient was α = 0.913.

#### Adaptation Difficulties in Returning to Routine Following COVID-19 Lockdown

Adaptation Difficulties in Returning to Routine Following COVID-19 Lockdown, is a new 6-item index that was developed for this study. In this novel index, participants were asked to rate how much they agreed with statements on a scale of 1 (“Strongly disagree”) to 5 (“Strongly agree”)—i.e., higher scores on the index represented more difficulties in returning to routine. Six statements were presented: “Although the days of social restrictions were difficult___” ___: (1) “… I miss the days of social restrictions;” (2) “… I have some concerns returning to routine;” (3) “…I would rather gradually return to routine;” (4) “…I wish I could stay at home for a longer time;” (5) “…I find it difficult to return to routine;” (6) “…I find it difficult to leave behind the days of social restrictions.” In this study, the Cronbach's coefficient was α = 0.845. A summary of the properties of this new measure is available in Table 1.

**Table 1:** Adaptation difficulties in returning to routine following COVID-19 scale.

			**Sum**	**Variance of sum**	**α**
	***M***	***sd***	**If item deleted**	**If item deleted**	**If item deleted**
1.	2.17	1.07	12.29	16.97	0.78
2.	2.44	1.15	12.02	16.75	0.79
3.	3.35	1.16	11.11	20.16	0.87
4.	2.27	1.13	12.19	16.04	0.76
5.	2.32	1.18	12.14	15.85	0.77
6.	1.91	1.03	12.55	16.55	0.76

*Scores are on a scale of 1 (“Strongly disagree”) to 5 (“Strongly agree”).*

*The six items are: “Although the days of social restrictions were difficult______”*

*1) “…I miss the days of social restrictions”.*

*2) “…I have some concerns returning to routine”.*

*3) “…I would rather gradually return to routine”.*

*4) “…I wish I could stay at home for a longer time”.*

*5) “…I find it difficult to return to routine”.*

*6) “…I find it difficult to leave behind the days of social restrictions”*

### Data Analysis

At the first stage, we examined the means for the study variables. Namely, depression: *M* = 4.44, range (0–17), *SD* = 3.87; return-to-routine adaptation difficulties: *M* = 2.42, range (1–5), *SD* = 0.81; and positive solitude: *M* = 3.69, range (1–5), *SD* = 0.76. We also examined the preliminary links between the study variables with Pearson's correlations (see Table 2 for means, standard deviations, and correlation for the study variables).

**Table 2:** Demographics and correlations for the study variables.

	***M/%***	***SD***	**1**	**2**	**3**	**4**	**5**	**6**
1. Depression^a^	4.44	3.87	-					
2. Adaptation difficulties	2.42	0.81	0.21^**^	-				
3. Solitude	3.69	0.76	−0.15^*^	0.01	-			
4. Age	61.57	12.81	−0.14^*^	−0.39^**^	−0.18^**^			
5. Gender^b^	75.8%	-	−0.13	−0.19^**^	−0.15^*^	0.20^**^		
6. Marital status^c^	79.20%	-	−0.30^**^	−0.01	0.15^*^	−0.05	0.21^**^	
7. Economic status^d^	3.88	0.88	−0.27^**^	−0.06	0.07	0.035	0.08	0.27^**^

*Total N = 293 (Regression included N = 234).*

a *Depression, PHQ-9.*

b *Gender, woman.*

c *Marital status, currently married, or living with a partner.*

d *Higher score (range 1–5) reflect better economic status.*

* *p < 0.05,*

** *p < 0.01*.

Subsequently, to examine our hypotheses, we conducted a multiple hierarchical linear regression analysis. Demographic variables (age, gender, marital status, and social economic status) were entered in Step 1. Level of return-to-routine adaptation difficulties and the moderator, positive solitude, were entered in Step 2. The interaction between level of return-to-routine adaptation difficulties and positive solitude was entered in Step 3. All predictors were mean-centered prior to moderation analysis. Significant interactions were probed with the PROCESS computational tool [V3.5; (44)]. This tool probes the significance of slopes at different levels of the moderator (i.e., positive solitude).

## Results

Based on the PHQ-9 cut-off score of ≥5, the sample demonstrated that 40.4% of the participants met the criteria of mild depressive symptoms and above, while 10.8% of the sample met the criteria of moderate to severe level of depressive symptoms, based on the PHQ-9 cut-off score of ≥10.

The median of *return-to-routine adaptation difficulties* was 2.33. In our sample, 44% reported moderate-to-high level of adaptation difficulties (≥2.50), whereas only a third of responders reported a low level (a score of <2) of adaptation difficulties.

As presented in Table 2, the level of return-to-routine adaptation difficulties was positively correlated with depressive symptoms (*r* = 0.21, *p* < 0.01). The level of positive solitude was negatively correlated with depressive symptoms (*r* = −0.15, *p* < 0.05). However, no significant correlation was found between the level of return-to-routine adaptation difficulties and the level of positive solitude. Return-to-routine adaptation difficulties, positive solitude and depressive symptoms were also negatively correlated with age (*r* = −0.14, *p* < 0.05; *r* = −0.39, *p* < 0.005; *r* = −0.18, *p* < 0.005, respectively).

Notably, older adults in our sample (age ≥ 65, *N* = 145) had lower rates of depressive symptoms (32.2%) than those of middle-aged adults (48.7%). Similarly, only 8.6% of the older adults in our sample reported a high level of return-to-routine adaptation difficulties (a score of ≥3) vs. 24.7% of middle-aged adults. Yet, for older adults the positive correlation between level of return-to-routine adaptation difficulties and depressive symptoms persisted (*r* = 0.38, *p* < 0.001).

The hierarchical regression analysis is presented in Table 3. It revealed that higher levels of return-to-routine adaptation difficulties were related to higher levels of depressive symptoms (β = 0.15, *t* = 2.34, *p* < 0.05). However, higher levels of positive solitude were related to lower levels of depressive symptoms (β = −0.15, *t* = −2.38, *p* < 0.05).

**Table 3:** Regression coefficients for the association between return-to-routine adaptation difficulties, positive solitude and depressive symptoms.

		**Depressive symptoms (PHQ-9)**	
	**Predictor**	***B (SE)***	**β**
**Step 1**	Age	−0.04^*^ (0.02)	−0.13
	Gender^a^	−0.35 (0.60)	−0.04
	Marital status^b^	−0.28^***^ (0.63)	−0.24
	SES^c^	−0.83^**^ (0.28)	−0.19
**Step 2**	Adaptation difficulties	0.72^*^ (0.31)	0.15
	Positive solitude	−0.76^*^ (0.32)	−0.15
**Step 3**	Adaptation difficulties × Positive solitude	−1.30^***^ (0.33)	−0.23
Total *R*^2^	0.23		

*Total N = 293 (Regression included N = 234).*

a *Gender, woman.*

b *Marital status, currently married, or living with a partner.*

c *Higher score (range 1–5) reflect better economic status.*

* *p < 0.05,*

** *p < 0.01,*

*** *p < 0.001*.

**Figure 1:**
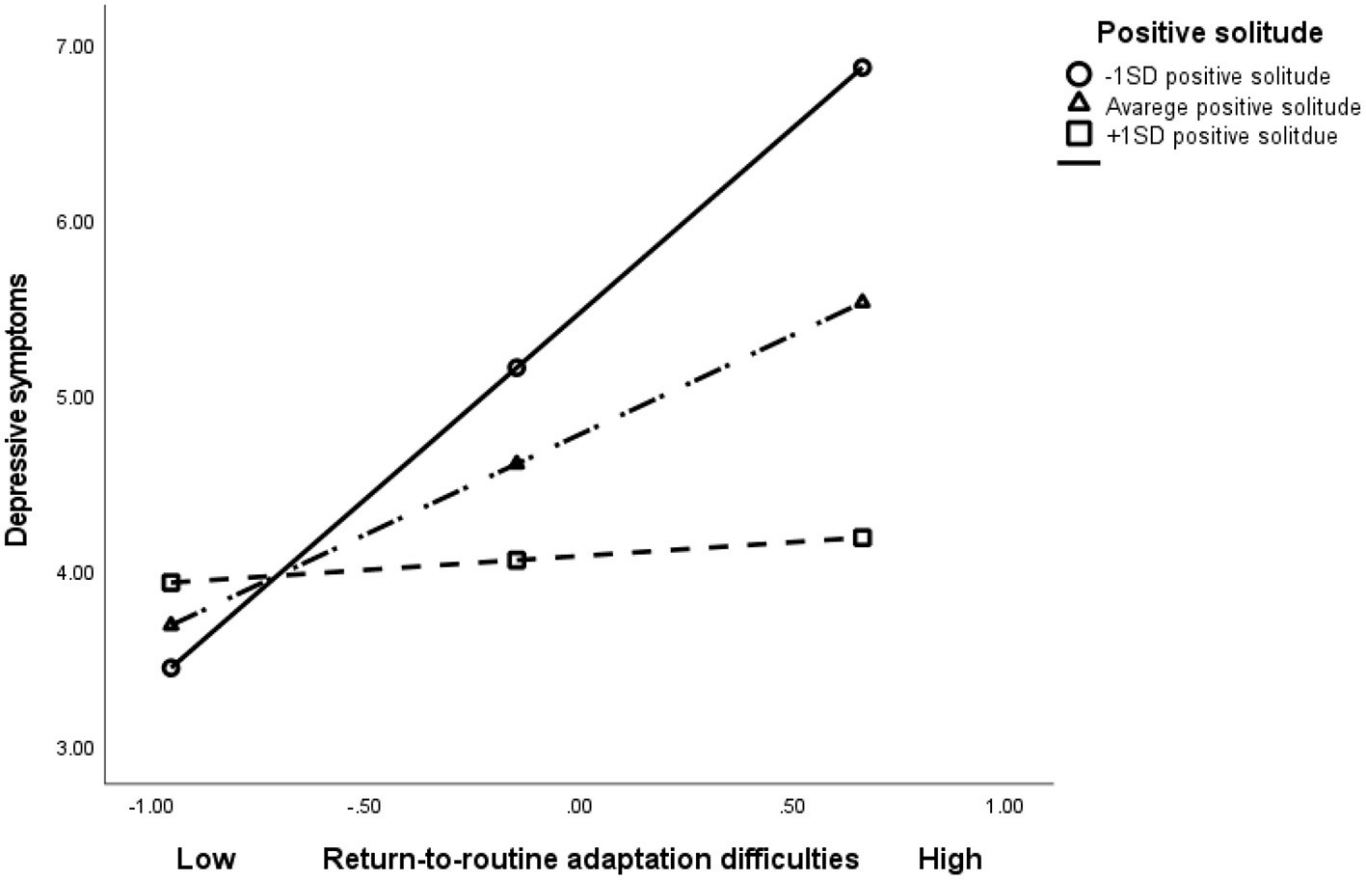
The association between return-to-routine adaptation difficulties, positive solitude and depressive symptoms.

The combination between level of return-to-routine adaptation difficulties and level of positive solitude was entered in the third step, revealing a significant interaction (β = −0.23, *t* = −3.95, *p* < 0.001), accounting for an additional 5% of the variance in depressive symptoms. The whole model explained 23.4% of the variance. Appling Hayes's (44) computational procedure showed that for individuals reporting low level of positive solitude (−1 SD), each additional return-to-routine adaptation difficulties score was associated with a significant increase of 1.62 points in level of depressive symptoms (*B* = 1.62, *t* = 4.31, *p* < 0.001)—i.e., the slope of return-to-routine adaptation difficulties × depressive symptoms was statistically significant. However, for individuals with a high level of positive solitude (+1 SD) each additional increase in return-to-routine adaptation difficulties was associated with an insignificant change in the level of depressive symptoms (*B* = −0.40, *t* = −0.97, *p* = *0.33*) (Figure 1).

## Discussion

The current study tested difficulties in adapting to routine following lifting of COVID-19 social restrictions and the cessation of a nation-wide lockdown in Israel. About half of the responders reported moderate-to-high levels of return-to-routine adaptation difficulties. These difficulties were positively correlated with depressive symptoms, while positive solitude was found to moderate this link. Namely, the association between return-to-routine adaptation difficulties and depression was mainly indicated for individuals with low positive solitude.

Given the global efforts to lift social restrictions, it is of interest to find that a large portion of adult individuals, express difficulties and concerns returning to normal life. For example, about half of our respondents agreed to some extent (provided a rating of 3 and above on a 1–5 scale) with the statement: “Although the days of social restrictions were difficult, I have some concerns returning to routine.” In other words, respondents were hoping to return to normal life, but now that social restrictions have been lifted, they express some anxiety. This seemingly paradoxical result confirms our hypothesis, indicating that the toll of social restrictions may have prolonged effects.

The current findings are of special interest to public welfare, as return-to-routine adaptation difficulties were associated with higher chance for clinical depressive symptoms. Note, for individuals with high positive experience while being by themselves (high positive solitude) the level of return-to-routine adaptation difficulties was not significantly associated with depressive symptoms. The finding supports the role of positive solitude as an efficient moderator in this period (30, 31). This should come of no surprise, as the main stressors during the COVID-19 pandemic are related to extreme social restrictions and lockdown (45).

In the current study, older adults report on less depressive symptoms than those reported by adults in general following COVID-19. This trend is in line with previous findings in the literature (11, 22). Interestingly, older adults have also reported fewer return-to-routine adaptation difficulties than have middle-aged adults. This may be related to retirement, as fewer older adults must return to work following the termination of social restrictions, and to increased resilience in older age [specifically emotional regulation; (46)]. Importantly, the link between adaptation difficulties and depressive symptoms persists in older age, indicating its strength across the adult life span.

Moreover, the ratio of individuals with mild (or more severe) depressive symptoms in our sample was very high, 40.4%, as compared to the pre- COVID-19 rate, 18.6%, found for Israeli adults (47). This extremely high proportion of depressive symptoms echoes other studies conducted during the pandemic across the globe (3, 11), pointing to the long-term negative effects of social restrictions. In addition, the literature indicates a link between life transitions and higher levels of depressive symptoms (48). Transitions, even from restrictions to improved conditions, might lead to psychological distress as indicated in our study. The large proportion of depressive symptoms that persist despite lifting social restrictions should lead policy makers to take actions incorporating clinical support on the national and personal levels, as part of the return to routine plan.

Finally, our analyses show that being able to enjoy spending time alone, as represented by high levels of positive solitude, was related to lower levels of depressive symptoms. It appears that these individuals are more resilient, not only during, but also after the end of a lockdown. More specifically, the moderation model indicates that higher levels of positive solitude could compensate for the deleterious outcomes of high levels of return-to-routine adaptation difficulties, and relate to lower levels of depressive symptoms. These findings support previous studies that present positive solitude as a potent capacity related to resilience (35).

### Limitations and Future Directions

This preliminary foray to the psychological cost of the transition to routine has several limits. The time sensitivity of the study (during the transition of Israel out of lockdowns) led to the choice of a cross-sectional and self-report design. This was also a convenience sample that may not represent the Israeli adult population. For example, 76% of our responders were women, possibly impacting the results [note, a higher proportion of female participants is not uncommon in this age group; e.g., (26, 49)]. The study was conducted in Israel with unique cultural aspects (51). Future studies may consider adapting our novel index to other languages and try to replicate the results in other countries and cultures (50), providing a more general statement regarding the association between return-to-routine adaptation difficulties and other indices of mental health. Moreover, due to the cross-sectional nature of the study, causality cannot be inferred. Thus, future studies should examine the long-term effects of social restrictions on mental health using additional cohorts, employing longitudinal and/or experimental designs.

## Conclusions

The current study offers a pioneering insight into the adaptation difficulties during the transition period from COVID-19 restrictions to routine. To the best of our knowledge, this is among the first studies to directly test post-lockdown psychological implications. The results point to the long-term effects of the pandemic on mental health issues, even when restrictions are lifted, and to positive solitude as a coping mechanism in time of stress (Figure 1). The current findings have global implications for clinicians as well as for governments, social organizations and other stakeholders. We hope the findings will raise awareness to adaptation difficulties returning to routine following social restrictions. We call policy makers to initiate programs informing the public on these issues. Simply put, it appears that negative psychological implications linger, even after the lockdown and associated restrictions have been lifted. In accordance, there is a need to develop accessible interventions and assessments, both *via* traditional face-to-face interactions and *via* tele-health platforms (7), to support a wide range of the population during social restrictions and upon return to routine. These interventions may wish to use positive solitude as a resource for coping during social isolation.

## Data Availability Statement

The raw data supporting the conclusions of this article will be made available by the authors, without undue reservation.

## Ethics Statement

The studies involving human participants were reviewed and approved by Reichman University, Herzliya. The patients/participants provided their written informed consent to participate in this study.

## Author Contributions

SK, YP, and BB-D contributed to the study design, data collection and analysis, and reporting and discussion. LR and AF contributed to the study design, data collection, reporting and review. All authors contributed to the article and approved the submitted version.

## Conflict of Interest

The authors declare that the research was conducted in the absence of any commercial or financial relationships that could be construed as a potential conflict of interest.

## Publisher's Note

All claims expressed in this article are solely those of the authors and do not necessarily represent those of their affiliated organizations, or those of the publisher, the editors and the reviewers. Any product that may be evaluated in this article, or claim that may be made by its manufacturer, is not guaranteed or endorsed by the publisher.
